# Estimated Impact of Targeted Pre-Exposure Prophylaxis: Strategies for Men Who Have Sex with Men in the United States

**DOI:** 10.3390/ijerph16091592

**Published:** 2019-05-07

**Authors:** Richard A. Elion, Mina Kabiri, Kenneth H. Mayer, David A. Wohl, Joshua Cohen, Anne C. Beaubrun, Frederick L. Altice

**Affiliations:** 1 George Washington University School of Medicine, Washington, DC 20009, USA; 2 Precision Health Economics, Los Angeles, CA 90025, USA; 3 The Fenway Institute, Harvard Medical School, Boston, MA 02215, USA; kmayer@fenwayhealth.org; 4 Chapel Hill School of Medicine, The University of North Carolina, Chapel Hill, NC 27514, USA; david_wohl@med.unc.edu; 5 Tufts University Institute for Clinical Research and Health Policy Studies, Tufts Medical Center, Boston, MA 02111, USA; joshua.cohen@tufts.edu; 6 Gilead Sciences, Foster City, CA 94404, USA; anne.beaubrun@gilead.com; 7 Section of Infectious Diseases, Yale University School of Medicine, New Haven, CT 06510, USA; frederick.altice@yale.edu

**Keywords:** pre-exposure prophylaxis (PrEP), men who have sex with men (MSM), agent-based model, simulation, human immunodeficiency virus (HIV), number needed to treat

## Abstract

Pre-exposure prophylaxis (PrEP) effectively reduces human immunodeficiency virus (HIV) transmission. We aimed to estimate the impact of different PrEP prioritization strategies among Black and Latino men who have sex with men (MSM) in the United States, populations most disproportionately affected by HIV. We developed an agent-based simulation to model the HIV epidemic among MSM. Individuals were assigned an HIV incidence risk index (HIRI-MSM) based on their sexual behavior. Prioritization strategies included PrEP use for individuals with HIRI-MSM ≥10 among all MSM, all Black MSM, young (≤25 years) Black MSM, Latino MSM, and young Latino MSM. We estimated the number needed to treat (NNT) to prevent one HIV infection, reductions in prevalence and incidence, and subsequent infections in non-PrEP users avoided under these strategies over 5 years (2016–2020). Young Black MSM eligible for PrEP had the lowest NNT (NNT = 10) followed by all Black MSM (NNT = 33) and young Latino MSM (NNT = 35). All Latino MSM and all MSM had NNT values of 63 and 70, respectively. Secondary infection reduction with PrEP was the highest among young Latino MSM (53.2%) followed by young Black MSM (37.8%). Targeting all MSM had the greatest reduction in prevalence (14.7% versus 2.9%–3.9% in other strategies) and incidence (49.4% versus 9.4%–13.9% in other groups). Using data representative of the United States MSM population, we found that a strategy of universal PrEP use by MSM was most effective in reducing HIV prevalence and incidence of MSM. Targeted use of PrEP by Black and Latino MSM, however, especially those ≤25 years, had the greatest impact on HIV prevention.

## 1. Introduction

Over 1.2 million individuals in the United States are infected with human immunodeficiency virus (HIV) [1]. While the overall number of people diagnosed with HIV has slowly decreased in the United States over the past decade [2], approximately 40,000 individuals are estimated to become HIV-infected each year.

Pre-exposure prophylaxis (PrEP) using tenofovir-based regimens—tenofovir disoproxil fumarate (TDF) and tenofovir alafenamide fumarate (TAF)—combined with emtricitabine (FTC) reduces HIV transmission; TDF/FTC is currently recommended for susceptible individuals at high risk of contracting HIV [3,4,5]. PrEP uptake has been slow, however, with approximately 120,000 persons prescribed PrEP [4]. This is a small fraction of the 1.1 million people estimated by the United States Centers for Disease Control and Prevention (CDC) to have indications for PrEP, of whom 73% are men who have sex with men (MSM) [6].

Racial disparities in the HIV epidemic among American MSM are evident. The prevalence of HIV in Black MSM was over 2-fold greater relative to White MSM in 2017 [7]. The HIV diagnosis rates were respectively ten and three times higher among Black and Latino MSM compared to White MSM [8]. Trends in new diagnoses of HIV infection have also been uneven by race/ethnicity. From 2010 to 2014, the number of annual new HIV infections declined by 11% among White MSM, but increased by 14% among Latino MSM and remained stable for Black MSM and for MSM as a total group [9]. Racial disparities are also present in the HIV continuum of care and PrEP access. Of the MSM with estimated indications for PrEP, 38% were Black and 27% were Latino [6], though data suggested that about 70% of individuals on PrEP were White [10,11]. According to data from the national HIV surveillance system, the percentage of MSM who had AIDS (acquired immunodeficiency syndrome) three years after HIV diagnosis was 8% higher among Black and Latino MSM than that among White MSM [12].

Existing predictive simulation studies in the United States (including agent-based models) have mainly assessed the impact of PrEP use among the entire MSM population [13,14,15,16,17,18,19,20], focusing on risk-behavior profiles rather than race and ethnicity. While some have studied the simulation of HIV infection among young Black or Latino populations, none has assessed the impact of PrEP use in these populations [21,22,23]. Furthermore, few have used the CDC recommended scoring system for PrEP indication when assessing PrEP impact [24]. To address some of these gaps, this study estimated the potential impact of different PrEP prioritization strategies among the high-risk Black and Latino MSM populations as identified by the CDC’s scoring system in the United States. This study also estimated the potential impact of targeting different age ranges within these two populations to identify the effectiveness of various prevention efforts, using a detailed simulation model.

## 2. Materials and Methods

We developed an agent-based model implemented in AnyLogic software (Supplement S1) to simulate the HIV epidemic among the MSM population in the United States, stratifying by age and race/ethnicity (Supplement S2). We also modeled risk level using the CDC’s seven-item HIV Incidence Risk Index (HIRI-MSM) (Table 1) to help identify and prioritize high-risk MSM for HIV prevention. The CDC considers an HIRI-MSM score of at least 10 as an indication for PrEP [24]. We simulated the impact of PrEP prioritization strategies on HIV transmission for different MSM populations.

An agent-based model is a flexible tool to simulate the dynamics of complex systems through modeling the interactions of discrete agents (individuals) in their environment. An important advantage of agent-based modeling over more traditional modeling techniques is the high degree of heterogeneity that can be introduced into a population based on risk [25]. The agent-based model of the MSM population and the impact of PrEP prioritization strategies on HIV transmission required various simulation components, such as the MSM population component, sexual contact network, HIV disease model, and a PrEP component, described as follows.

### 2.1. MSM Population Component

The MSM population component characterized the nationally representative MSM population in the United States. MSM were defined as agents, autonomous entities in the simulation with certain heterogeneous characteristics and attributes that diversely defined their sexual partnership and HIV disease status during the simulation period. Important sources of heterogeneity considered for the MSM population were age, race/ethnicity, relationship types and duration, sexual positioning preferences, and preferential age and race/ethnicity mixing.

We included detailed population characteristics such as age and race/ethnicity, and incorporated the matched HIV prevalence. Parameters were largely based on previously published literature as described in Table 2. Individuals were added to and removed from the model according to birth [26] and mortality rates [27]. The model applied standardized mortality ratios for HIV-infected MSM relative to the general population [28], which varied by HIV disease stage and viral suppression status.

### 2.2. Sexual Contact Network

The sexual contact network component incorporated rules to stochastically create and maintain a network of MSM according to their sexual mixing patterns and preferences over the simulation period. Individuals entered the sexual contact network at the age of sexual onset [26] and became eligible to form casual and regular partnerships (Table 2). Casual partnerships represented a single sexual encounter between two individuals. The desired frequency at which these encounters occurred was different for all individuals in the model [39]. Regular partnerships represented steady relationships, characterized mainly by their duration [35] and frequency of sexual intercourse [39]. The distribution of partnership types, regular partnership duration, and dissolution rates are presented in Table 2. Individuals could be involved in multiple casual and/or regular partnerships at any time [34] (Supplement S2).

We included assortative sexual mixing patterns based on race/ethnicity, age, sero-status (status of either having or not having detectable HIV antibodies) preferences (Supplement S2), and sexual positioning preferences (Table 2), in the partnership formation process. The details of assortative sexual mixing patterns are included in Supplement S2.

The status of MSM sexual partnerships and the frequency of sexual acts per partnership were updated every day in the simulation. The baseline per-act infection risk for HIV serodiscordant partnerships was 0.0011 for an HIV-negative insertive MSM and 0.0138 for an HIV-negative receptive MSM [43]. Circumcision reduced the probability of infection by 27% [44]. The probability of HIV transmission per sexual act was also adjusted according to the HIV stage and viral load suppression status of the infected partner, and condom use. Condom use depended on the individual’s risk category for sexual encounters, which was assigned based on race, age, and substance use status (Supplement S2). We assumed that all low-risk MSM used condoms [38]. We also assumed that the probability of condom use among high-/moderate-risk HIV-negative MSM was 39% and 45% for receptive and insertive sexual contact, respectively [39]. The structure and other parameters used in the calculation of per-act infection risk are presented in more detail in Supplement S2.

### 2.3. HIV Disease Model

**Figure 1:**
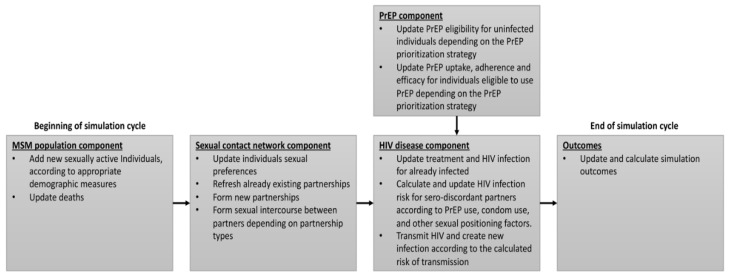
Summary of updates in model components during each simulation cycle. Note: model cycle was defined as a day. Abbreviations: HIV = human immunodeficiency virus; MSM = men who have sex with men; PrEP = pre-exposure prophylaxis; AIDS = acquired immune deficiency syndrome.

The HIV disease model stochastically simulated HIV disease transmission, progression, and treatment for infected MSM. For HIV-positive MSM, further heterogeneity was modeled through an interplay between HIV stage, HIV screening, retention in care, and viral suppression status. For HIV-uninfected individuals, additional sources of heterogeneity included PrEP eligibility, uptake, and adherence. The status of each agent was updated for every simulation cycle (every day) according to these components over the simulation period (Figure 1).

Our model included three stages of HIV infection: acute, chronic (clinical latency), and advanced stage (AIDS). In the absence of treatment, we assumed that individuals spent an average of 52 days with a high viral load in the acute stage (Supplement S3) [45]. Viral load decreased at the end of the acute stage and the start of the chronic stage, to a viral set point. We assumed that the chronic stage typically lasted about 10 years [46], a period characterized by a slow depletion of CD4 lymphocytes and increasing viral load. The final stage (AIDS), characterized by high viral load and low CD4 count, lasted up to three years, after which untreated individuals inevitably died.

In the absence of PrEP, we included screening for HIV-uninfected individuals according to different screening rates (Table 2). After a positive HIV test, individuals were considered diagnosed and started treatment, with race-dependent probability of retention in care [42] (Table 2). HIV-infected individuals on treatment transitioned in a stochastic manner between a suppressed and an unsuppressed viral load state based on monthly probabilities of suppression and rebound experienced by patients on HIV antiretroviral therapy (ART) [41,42]. Individuals not retained in care followed the disease progression of untreated individuals. Irrespective of diagnosis status and spontaneous HIV testing, the model allowed for individuals who progressed to AIDS to be diagnosed and automatically retained in care at a certain rate. The details of HIV disease model parameters are provided in Supplement S3.

### 2.4. PrEP Component

#### 2.4.1. PrEP Prioritization Scenarios

The PrEP component incorporated the impact of PrEP on disease transmission through details of PrEP uptake and effectiveness. We designed prioritization scenarios to compare the impact of prioritizing PrEP use for all MSM versus Black and Latino MSM only, considering the HIV risk index for each uninfected individual based on the CDC HIRI-MSM score (Table 1) [24]. In the model, uninfected MSM were eligible to receive PrEP according to their HIRI-MSM score if they satisfied the following criteria, each defined as a prioritization scenario: MSM with HIRI-MSM score of ≥10 (MSM-10+), all Black MSM with HIRI-MSM score of ≥10 (BMSM-10+), young (≤25 years) Black MSM with HIRI-MSM score of ≥10 (YBMSM-10+), all Latino MSM with HIRI-MSM score of ≥10 (HMSM-10+), and young (≤25 years) Latino MSM with HIRI-MSM score of ≥10 (YHMSM-10+). Under each prioritization scenario, PrEP eligibility was evaluated for uninfected individuals and updated every six months for previously ineligible individuals by updating the HIRI-MSM score. We also simulated a No-PrEP counterfactual scenario in order to estimate HIV-related outcomes without the impact of PrEP.

#### 2.4.2. PrEP Uptake, Adherence, and Effectiveness

Under each prioritization scenario, we used a log-linear regression model [47] to assign PrEP uptake status to the eligible individuals. Adherence, including treatment interruption and permanent discontinuation, was considered the main driver of effectiveness in decreasing HIV infection risk. We obtained real-world PrEP uptake and adherence distributions, dependent on individual characteristics, from the United States PrEP Demonstration Project (PrEP Demo) [47,48] and from the iPrEx open-label extension (OLE) study [45]. Additional details on PrEP adherence and effectiveness are presented in Supplement S4.

PrEP use impacted the sexual network structure. Under all prioritization scenarios, the network structure was preserved from the beginning of the simulation until 2016, when we started to model the impact of PrEP on reducing HIV transmission. After 2016, the sexual network component was different only among MSM with prevented infection, compared to a no-PrEP scenario. PrEP use changed the pool of MSM with different sero-statuses over time depending on the prioritization scenario, resulting in different network structure since sero-sorting preferences affected partnership formation.

### 2.5. Model Outcomes

Under each prioritization scenario, we estimated the number needed to treat (NNT) with PrEP among uninfected individuals to prevent one new HIV infection during the period from 2016 through 2020. Under each PrEP prioritization scenario, we calculated NNT as the person-time on PrEP divided by the number of infections avoided compared to the No-PrEP counterfactual scenario. To better understand PrEP within the overall context of preventive health measures, we compared the PrEP NNT value to the NNT calculated for statins to prevent primary myocardial infarction [49]. We used statins as benchmark since statins are commonly used as a preventative measure for a fairly highly prevalent disease, which also has a behavioral component to the risk (e.g., diet and exercise), similar to PrEP use for high risk MSM to prevent HIV and AIDS.

We projected the percentage of total MSM eligible to receive PrEP, uptake rate within the eligible category, and the average per-person time on PrEP as an indicator for PrEP adherence. We also estimated changes in the HIV point prevalence among all MSM, HIV incidence rate among PrEP users, and the number of secondary infections (new infections among all MSM non-PrEP users) avoided in 2016–2020.

### 2.6. Model Calibration

Using an iterative process, we calibrated the distribution of patients by age and race/ethnicity, and the distribution of the number of regular partnerships among MSM over time prior to the year 2010, to accurately represent the prevalence of HIV among MSM by race/ethnicity during 2010–2013, according to published data by the CDC [33] (Supplement S5).

## 3. Results

During the 5-year projection period from 2016–2020, the percentage of PrEP-eligible MSM varied greatly across different prioritization strategies, with MSM-10+ (51% eligible) and YBMSM-10+ (2% eligible) scenarios yielding the highest and lowest percentages of PrEP-eligible MSM, respectively (Table 3). The proportion of PrEP uptake among the eligible MSM, however, was similar between different scenarios, ranging from 66% to 74%. Our simulation model results prior to the projection period (2016–2020) simulated the HIV epidemic using historic data in 2010–2013 (Supplement S6).

**Figure 2:**
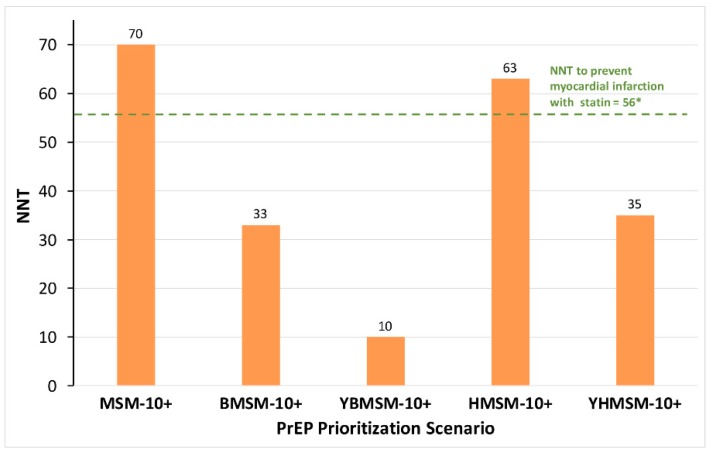
Number needed to treat to prevent one new HIV infection under each PrEP prioritization scenario in 2016–2020. * NNT estimate based on a study by Taylor et al. [49]. Abbreviations: NNT = number needed to treat (to prevent one infection); PrEP = pre-exposure prophylaxis; HIRI-MSM = incidence risk index for MSM; MSM-10+ = PrEP prioritization scenario targeting all MSM with HIRI-MSM score of ≥10; BMSM-10+ = PrEP prioritization scenario targeting Black MSM with HIRI-MSM score of ≥10; YBMSM-10+ = PrEP prioritization scenario targeting young (≤25 years) Black MSM with HIRI-MSM score of ≥10; HMSM-10+ = PrEP prioritization scenario targeting Latino MSM with HIRI-MSM score of ≥10; YHMSM-10+ = PrEP prioritization scenario targeting young (≤25 years) Latino MSM with HIRI-MSM score of ≥10.

**Figure 3:**
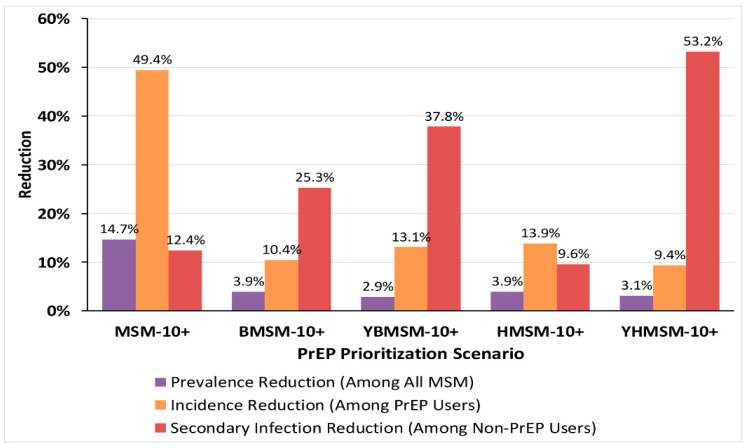
Reduction in the HIV prevalence, incidence, and secondary infections under each PrEP prioritization scenario in 2016–2020. Note: Estimated results are compared to the No-PrEP scenario. Abbreviations: NNT = number needed to treat (to prevent one infection); PrEP = pre-exposure prophylaxis; HIRI-MSM = incidence risk index for MSM; MSM-10+ = PrEP prioritization scenario targeting all MSM with HIRI-MSM score of ≥10; BMSM-10+ = PrEP prioritization scenario targeting Black MSM with HIRI-MSM score of ≥10; YBMSM-10+ = PrEP prioritization scenario targeting young Black MSM (≤25 years) with HIRI-MSM score of ≥10; HMSM-10+ = PrEP prioritization scenario targeting Latino MSM with HIRI-MSM score of ≥10; YHMSM-10+ = PrEP prioritization scenario targeting young Latino MSM (≤25 years) with HIRI-MSM score of ≥10.

The NNT was lower for PrEP prioritization strategies focusing on Latino MSM (NNT = 63) compared to all MSM (NNT = 70, Figure 2), but prioritizing PrEP use for Black MSM (NNT = 33), young Black MSM (NNT = 10), and young Latino MSM (NNT = 35) yielded even lower NNT values. Offering PrEP to all MSM with HIRI-MSM ≥10 (MSM-10+) yielded the largest reduction in the prevalence of HIV among all MSM (14.7%) between 2016 and 2020, and decline in incidence of HIV among PrEP users (49.4%) (Figure 3). For other scenarios, the reductions in the prevalence of HIV (2.9%–3.9%) and in the incidence of HIV (9.4%–13.9%) among PrEP users were lower than those estimated in MSM-10+ scenario. The cumulative number of new infections in the MSM population during 2016–2020 under the MSM-10+ scenario decreased by 50% compared to the No-PrEP scenario (Table 3). Compared to the No-PrEP scenario, the cumulative number of new infections in the MSM population during 2016–2020 decreased by 16% under HMSM-10+, by 14% under BMSM-10+, and by 8% under YBMSM-10+ and YHMSM-10+ scenarios.

During 2016 to 2020, the decrease in the cumulative number of secondary infections—new infections among non-PrEP-using MSM—was the highest when targeting young Black and Latino populations for PrEP; reductions were projected at 53% under the YHMSM-10+ and at 38% under the YBMSM-10+ scenarios in 2016–2020 relative to the No-PrEP scenario (Figure 3). Under the BMSM-10+ scenario, the cumulative number of secondary infections avoided decreased by 25%, ranking third highest in its impact, whereas the decrease was only 12% under the MSM-10+ scenario and 10% under the HMSM-10+ scenario for the same period relative to the No-PrEP scenario.

## 4. Discussion

We developed an agent-based simulation that included social network dynamics in various MSM populations and incorporated risk behavior, sexual partnerships, and other HIV transmission factors. We found that PrEP use by young Black MSM was the most efficient strategy in preventing HIV infection, with ten young Black MSM needing to be treated with PrEP to prevent one new HIV infection. For every 33 Black MSM of any age using PrEP, one new HIV infection would be averted. The model suggested slightly higher NNT for Latino MSM, with a greater effect seen among younger Latino MSM. These estimated NNT values for PrEP use among MSM of color are lower than the NNT of statins to prevent primary myocardial infarction (NNT = 56) [49], and suggest that PrEP strategies focused on Black and Latino MSM, especially young individuals, are a highly efficient HIV prevention approach.

Not unexpectedly, universal PrEP uptake by all MSM would have the greatest effect on reducing HIV incidence and prevalence overall, but at a substantially increased NNT compared to more targeted use of PrEP among those at greater behavioral and/or epidemiological risk. PrEP use strategies that targeted young Black and Latino MSM, however, resulted in higher percentages of secondary infections avoided.

Our study underscores and supports the significance of targeted strategies for PrEP use and aligns with the HIV prevention recommendations from other published sources [2,18]. According to the CDC, young Black and Latino MSM are at the greatest risk of acquiring the disease. Investment in HIV prevention programs for these populations would be expected to be the most efficient and practical approach to control HIV transmission in the United States among MSM.

These results also align with other modeling studies that find the greatest benefit in reducing the number of infections with targeted high-risk populations, particularly young individuals [13,15,17,50]. There are several strengths of our analyses. Our model quantifies the impact of different PrEP-focused HIV prevention strategies for different populations at risk using innovative methods. Most prior studies addressing similar research questions have centered on the cost-effectiveness of PrEP utilization strategies mainly for all MSM populations. To our knowledge, this is the first study to not only evaluate subgroups of high-risk populations previously unassessed, but also to use the CDC’s HIRI-MSM scoring to identify risk and calculate outcomes such as prevalence and incidence proportions to show the impact of PrEP in such populations. We compared different strategies using NNT and secondary infections averted, which provide a better understanding of the overall impact of these strategies on the HIV epidemic and the general MSM population. Moreover, we developed an agent-based simulation model, in which the characteristics and behavior of individuals, rather than populations or subgroups, are modeled. This means that the characterization of the sexual contact network and HIV transmission emerge from the bottom up, that is, individual characteristics and sexual behaviors drive the composition of the sexual contact network, which allows for the inclusion of different aspects of population heterogeneity.

There are also limitations to our study that should be considered when interpreting these results. First, the approach used did not include the impact of HIV transmission within the heterosexual population or people who inject drugs among subpopulations of MSM, which could minimally underestimate the number of new HIV infections. Second, we assumed that overall sexual behavior trends among MSM did not change through 2020. Third, we did not model White, Asian/Pacific Islander, or other ethnicities discretely. Fourth, we relied on data from the literature for the historic ratios of undiagnosed HIV infections. These estimates, however, could be biased, thereby leading to under or overestimation of the impact of PrEP on model outcomes. Finally, we used the CDC’s HIRI-MSM score of ≥10 for PrEP indication. HIRI-MSM score did not include the impact of condom use, older ages, and how different regions of the United States are affected by HIV/AIDS on treatment indication. These factors were not of significance to include in the process of developing HIRI-MSM scoring criteria based on the specific MSM sample used, though their exclusion might artificially under-represent MSM risk pool among MSM who do not use condoms or in areas with a higher HIV prevalence. Although we followed CDC’s guidelines for PrEP indication according to the HIRI-MSM score, we included the impact of condom use, age, and race in calculating the risk of HIV transmission between MSM. Our study focused on the general MSM population and we did not adjust for geographical regions.

The results of our study are informative for policy makers and signify the centrality of young Black and Latino MSM in the efforts to stem HIV transmission and PrEP uptake strategies. While treating all high-risk MSM has the greatest impact on changing the disease burden of HIV in the United States, it also requires treating more individuals and a greater use of resources. Our results suggest that when prioritization is necessary due to limited expenditures or capacity concerns, focusing first on young Black and Latino MSM could be the most efficient step towards accruing significant health gains from a strategic PrEP prevention effort.

Our results highlight the need to reduce racial disparities in access to PrEP and HIV continuum of care. Black and Latino MSM are among the most challenging groups for the scale-up of PrEP. Barriers could include limited access to quality healthcare, poverty, lack of or poorer health insurance coverage, and higher rates of incarceration [51,52,53]. A study by Oster et al., however, found that similar rates of newly diagnosed Black and White MSM had health insurance, saw a provider, and received HIV screening tests [54]. Other studies confirmed that although socio-economic disparities, particularly lower education and higher poverty and unemployment, could be sources of health disparities, they do not entirely explain the differences between levels of care among Black and Latino MSM compared to White MSM [54,55,56,57]. These researchers found that disparities are also caused by cultural factors such as racial/sexual discrimination, stigma, inadequate knowledge related to HIV/AIDS risk and prevention measures, and overall relationship to health. MSM of color are less likely to know the HIV sero-status of their partners before sexual encounters and have longer periods of infectiousness since they are significantly less likely to seek HIV screening and care [54]. These cultural/behavioral factors combined with racial assortative mixing patterns would increase partner pool risk and in turn HIV transmission among Black and Latino MSM. Altogether, addressing racial and health disparities is a complex issue. Policies to reduce obstacles to effective HIV prevention modalities should involve developing a deeper understanding of these disparities and promote cultural and behavioral interventions along with medical prevention methods, particularly for younger MSM of color.

## 5. Conclusions

In conclusion, our modeling demonstrates and quantifies the benefits of focusing HIV prevention by using PrEP in populations at greatest risk. The NNT to prevent new HIV infection among these groups is lower than for many routinely prescribed health measures. If prioritization for financial or system concerns is required, PrEP prioritization strategies should focus on Black and Latino MSM, especially younger individuals, with barriers to such use reduced or eliminated.

## Figures and Tables

**Table 1:** Seven-item CDC HIV incidence risk index for MSM (HIRI-MSM).

HIRI-MSM Risk Index [24]			
1.	How old are you today (years)?	<18 years	Score 0
		18–28 years	Score 8
		29–40 years	Score 5
		41–48 years	Score 2
		≥49 years	Score 0
2.	How many men have you had sex with in the last 6 months?	>10 male partners	Score 7
		6–10 male partners	Score 4
		0–5 male partners	Score 0
3.	In the last 6 months, how many times did you have receptive anal sex (you were the bottom) with a man?	1 or more times	Score 10
		0 times	Score 0
4.	How many of your male sex partners were HIV-positive?	>1 positive partner	Score 8
		1 positive partner	Score 4
		<1 positive partner	Score 0
5.	In the last 6 months, how many times did you have insertive anal sex (you were the top) with a man who was HIV-positive?	5 or more times	Score 6
		0 times	Score 0
6.	In the last 6 months, have you used methamphetamines such as crystal or speed?	Yes	Score 5
		No	Score 0
7.	In the last 6 months, have you used poppers (amyl nitrate)?	Yes	Score 3
		No	Score 0
The total of entries in right column is the calculated score.			Total Score

Abbreviations: CDC = Centers for Disease Control and Prevention; HIV = human immunodeficiency virus; MSM = men who have sex with men.

**Table 2:** HIV epidemic and transmission risk factors among MSM in the United States

Model Parameter	Value				Source
MSM population (estimated during 2009 to 2013)	4,503,080 (3.9% of the United States population)				[29,30]
Distribution of race/ethnicity among MSM (%)					[31]
Black	8.9				
White	71.4				
Latino	15.9				
Other	3.8				
MSM age distribution by race/ethnicity (%)	Black	White	Latino	Other	[32]
18–19	4.5	2.2	4.4	2.9	
20–29	34.2	24.8	39.8	44.2	
30–39	23.8	20.4	28.0	30.9	
40–49	25.9	28.5	21.1	16.1	
50–59	10.1	18.2	5.6	5.7	
60+	1.5	5.9	1.1	0.2	
Prevalence of HIV among the MSM population (average during 2009 to 2013)	448,026 (10% of the MSM population)				[33]
Distribution of race/ethnicity among HIV-infected MSM (average during 2009 to 2013) (%)					[33]
Black	29.9				
White	43.9				
Latino	20.7				
Other	5.5				
Standardized mortality ratios for different HIV stages					[28]
Acute stage, suppressed or unsuppressed viral load	1				
Chronic Stage, diagnosed, suppressed viral load	1.05				
Chronic Stage, diagnosed, unsuppressed viral load	2.06				
Chronic Stage, undiagnosed, unsuppressed viral load	3.51				
Early final stage (AIDS)	23.4				
End final stage (AIDS)	24.9				
Mean age of sexual onset (years)					[26]
Black	16.4				
Latino	16.3				
White	17.4				
Other	16.9				
Distribution for the desired types of partnerships					[34]
Casual	14.6%				
Regular	51.1%				
Both	34.3%				
Distribution for the duration of regular partnership					[35]
Short-term	64%				
Long-term	36%				
Rate of regular partnership dissolution					[35]
Short-term	0.38565				
Long-term	0.09977				
Distribution for the positional preferences					
Insertive	30.3%				[36]
Receptive	14.2%				
Versatile	55.4%				
Proportion of MSM with circumcision by race					[37]
Black	0.757				
White	0.908				
Latino	0.440				
Other	0.440				
Rate of condom use by risk group					
Low-risk MSM	100%				[38]
High-risk MSM—receptive intercourse with HIV-infected partner	39%				[39]
High-risk MSM—insertive intercourse with HIV-infected partner	45%				[39]
Testing frequency categories for HIV-uninfected MSM					[40]
Never	20.6%				
High frequency	64.3%				
Low frequency	15.1%				
Testing rate for the high-frequency category	0.00509				[40]
Testing rate for the low-frequency category	0.00061				
Proportion of MSM who remained in care upon HIV diagnosis					
Non-Black MSM	0.40000				[41]
Black MSM	0.31596				[41,42]

Abbreviations: HIV = human immunodeficiency virus; MSM = men who have sex with men; PrEP = pre-exposure prophylaxis; AIDS = acquired immune deficiency syndrome.

**Table 3:** Percentage of MSM eligible for PrEP under each prioritization scenario, and percentage of uptake and average time on PrEP within each target group in 2016–2020.

PrEP Prioritization Scenario	Eligibility Among All Noninfected MSM (%)	PrEP Uptake Among Eligible	Average Per-Person Time on PrEP (Years)	Reduction in Total New Infections ^1^
**MSM-10+**	51%	73%	3.00	50%
**BMSM-10+**	5%	67%	2.38	14%
**YBMSM-10+**	2%	66%	2.50	8%
**HMSM-10+**	13%	74%	2.97	16%
**YHMSM-10+**	5%	69%	2.99	8%

^1^ Compared to the No-PrEP scenario. Abbreviations: MSM = men who have sex with men; PrEP = pre-exposure prophylaxis; HIRI-MSM = incidence risk index for MSM; MSM-10+ = PrEP prioritization scenario targeting all MSM with HIRI-MSM score of ≥10; BMSM-10+ = PrEP prioritization scenario targeting Black MSM with HIRI-MSM score of ≥10; YBMSM-10+ = PrEP prioritization scenario targeting young (≤25 years) Black MSM with HIRI-MSM score of ≥10; HMSM-10+ = PrEP prioritization scenario targeting Latino MSM with HIRI-MSM score of ≥10; YHMSM-10+ = PrEP prioritization scenario targeting young (≤25 years) Latino MSM with HIRI-MSM score of ≥10.

## Supplementary Materials

The following supplementary materials are available online at https://www.mdpi.com/1660-4601/16/9/1592/s1, Supplement S1: Model Implementation; Supplement S2: Details of Sexual Contact Network; Supplement S3: Details of HIV Disease Component; Supplement S4: Details of Pre-Exposure Prophylaxis (PrEP) Component; Supplement S5: Model Calibration and Validation; Supplement S6: Model Validation Results.
