# Supplementary material for: Estimated Impact of Targeted Pre-Exposure Prophylaxis: Strategies for Men Who Have Sex with Men in the United States
Source: Int J Environ Res Public Health. 2019 May 7;16(9):1592. doi: 10.3390/ijerph16091592 (PMC6539923; doi:10.3390/ijerph16091592)
Supplement: Supplementary file 1 [file ijerph-16-01592-s001.pdf]

Supplement

This Supplement provides data and supporting results, including validation, sensitivity analyses, and additional clinical scenarios for the first section.

Table of Contents

Supplement S1. Model Implementation **2**
Supplement S2. Details of Sexual Contact Network **3**
Partnership Types and Partnership Formation 3
Sexual Mixing Patterns 3
Sexual Intercourse 5
Supplement S3. Details of HIV Disease Component **7**
HIV screening 7
Supplement S4. Details of Pre-exposure prophylaxis (PrEP) Component **8**
Supplement S5. Model Calibration and Validation **10**
Supplement S6. Model Validation Results **11**
References **13**

Supplement S1. Model Implementation

The model was implemented in AnyLogic, a commercially available simulation software that allows for the integration of various modeling paradigms (e.g. agent-based modeling, system dynamics modeling and discrete event modeling) into a single software.

Supplement S2. Details of Sexual Contact Network

Partnership Types and Partnership Formation

Detailed parameters for assigning the number of casual or regular partnerships are presented in **Table 1**.

Table 1. Sexual Contact Network Parameters

Parameter	Value	Source
**Estimated log-normal model for the number of casual partners in the last 6 months, stratified by race**		(1)
Intercept	2.00134	
Black MSM (indicator variable)	-0.30713	
Hispanic (indicator variable)	-0.04513	
White (indicator variable)	0.13855	

Abbreviation: MSM = men who have sex with men.

Sexual Mixing Patterns

We considered the compatibility of individuals in terms of racial, age, sexual positioning and sero-status preferences, in the formation of new partnerships. Four distinct racial classes were included in the model: White, Black, Hispanic, and other (2). Both racial (**Table 2**) and age mixing (**Table 3**) preferences were race-dependent (1, 3).

Sexual positioning preferences were divided into three classes: insertive, receptive and versatile anal intercourse (4). Individuals who had an insertive (receptive) sexual positioning preference could form partnerships with individuals whose sexual positioning preference was either receptive (insertive) or versatile. Individuals who had a versatile sexual positioning preference could form partnerships with all individuals, irrespective of their sexual positioning preference.

The probability of an individual being a sero-sorter or not was both race and human immunodeficiency virus (HIV) infection status dependent (5). If neither of two individuals were a sero-sorter, a partnership could be formed, irrespective of the HIV infection sero-status of the individuals. If one or both of the individuals were sero-sorters, a relationship could only be formed if both individuals had the same presumed sero-status. The proportion of sero-sorters among HIV-negative and HIV-positive MSM by race are presented in Table 4.

Table 2. Race mixing distribution for regular and casual partnerships

	Black	White	Hispanic	Other	Source
**Black**	78.2%	8.7%	2.6%	52.9%	(3)
**White**	8.1%	73.9%	27.3%	20.6%	
**Hispanic**	11.7%	15.9%	68.8%	23.5%	
**Other**	2.0%	1.5%	1.3%	3.0%	

Table 3. Age mixing patterns among MSM by race

Parameters	Value (%)					Source
**Black MSM**		age <25	age 25–34	age 34–44	age 45+	(1)
	age <25	44	24	19	16	
	age 25–34	40	48	36	24	
	age 34–44	15	24	38	34	
	age 45+	2	4	7	26	
**White MSM**		age <25	age 25–34	age 34–44	age 45+	
	age <25	50	26	21	21	
	age 25–34	40	52	44	32	
	age 34–44	8	20	32	36	
	age 45+	2	2	4	12	
**Hispanic MSM**		age <25	age 25–34	age 34–44	age 45+	
	age <25	41	19	15	13	
	age 25–34	42	49	39	28	
	age 34–44	12	24	32	36	
	age 45+	5	8	13	23	
**Other MSM**		age <25	age 25–34	age 34–44	age 45+	
	age <25	41	23	10	6	
	age 25–34	42	30	25	12	
	age 34–44	12	33	44	45	
	age 45+	5	13	21	37	

Abbreviation: MSM = men who have sex with men.

Table 4. Sero-sorting preferences by race.

Race	Intentional sero-sorting partnerships	Source
**HIV-positive MSM**		**(6)**
Black	15.3%	
White	12.2%	
Hispanic	26.1%	
Other	13.5%	
**HIV-negative MSM**		
Black	45.9%	
White	52.1%	
Hispanic	47.6%	
Other	57.3%	

Sexual Intercourse

Sexual Frequency

In casual partnerships, only one sexual act occurred per partnership, though various acts could occur with different partners on the same day. In regular partnerships, various sexual acts occurred over time with the same partner, with a higher sexual act frequency during the first month as compared to the rest of the partnership (1).

Infection Risk

The base per-sexual-act HIV infection risk for an uninfected individual represented the probability of infection from a single sexual act with an infected individual in the unsuppressed chronic stage of HIV infection, without the presence of preventive measures (**Table 5**). This base infection risk depended on the sexual position (insertive/receptive) of the infected individual during intercourse (7).

We modified the base infection risk according to the HIV disease stage and suppression status of the infected partner, circumcision, condom use and the presence of other sexually transmitted diseases (STDs) (**Table 5**). The risk of infection increased when the infected partner was in the acute or final stage of HIV infection (8). The risk also increased with the presence of STDs (9). The risk of infection decreased, on the other hand, due to circumcision (7), condom use (10), or when the viral load of the HIV-infected partner was suppressed (11).

Condom use

Condom use depended on individual's risk category for sexual encounters, which was assigned based on race, age, and substance use status (**Table 5**). We assumed that all low-risk MSM used condoms (12). We also assumed that the probability of condom use among high/moderate risk HIV-negative MSM was 39% and 45% for receptive and insertive sexual contact, respectively (1). Upon sexual intercourse with a condom within a sero-discordant partnership, a reduction factor was applied to the base per-sexual-act HIV infection risk. This reduction factor depended both on race and sexual position of the HIV infected individual (10, 13) (**Table 5**).

Table 5. Parameters for the sexual transmission of HIV infection

Parameters	Value	Source
**Multinomial model to assign the risk class of MSM, as a function of age, race and substance use**		(12)
Intercept high risk	0.938	
Age high risk	-0.083	
Non-injection drug user high risk	0.693	
White high risk	1.300	
Intercept moderate risk	-0.062	
Age moderate risk	0.74	
Non-injection drug user moderate risk	0.255	
White moderate risk	1.364	
**Baseline per act infection risk and associated risk factors**		
Baseline per act infection risk for HIV- that is insertive, having anal sex with HIV+ without preventive measures	11.0/10000	(7)
Baseline per act infection risk for HIV- that is receptive, having anal sex with HIV+ without preventive measures	138.0/10000	(7)
Multiplicative risk factor to increase per act infection risk during HIV acute stage	26.000	(8)
Multiplicative risk factor to decrease per act infection risk if circumcised	0.2700	(7)
Multiplicative risk factor to decrease per act infection risk if condom used (Non-Black population) - insertive	0.3710	(10)
Multiplicative risk factor to decrease per act infection risk if condom used (Black MSM) - insertive	0.5165	(13)
Multiplicative risk factor to decrease per act infection risk if condom used (Non-Black population) - receptive	0.2770	(10)
Multiplicative risk factor to decrease per act infection risk if condom used (Black MSM) - receptive	0.6218	(13)
Multiplicative risk factor to decrease per act infection risk if condom used (Non-Black population) - versatile	0.2950	(10)
Multiplicative risk factor to decrease per act infection risk if condom used (Black MSM) - versatile	0.6009	(13)
Multiplicative risk factor to increase per act infection risk during final phase (AIDS)	7.0000	(8)
Multiplicative risk increase factor for per act infection risk in the presence of STD (irrespective of STD being on HIV+ or HIV-)	3.1310	(9)
Multiplicative risk factor to decrease per act infection risk if infection is suppressed	0.0000	(11)

Abbreviation: MSM = men who have sex with men; AIDS = acquired immune deficiency syndrome; STD = sexually transmitted disease.

Supplement S3. Details of HIV Disease Component

HIV screening

Presumed HIV sero-negative individuals were divided into three classes, depending on their HIV testing frequency. A certain percentage of individuals never tested for HIV. Other were divided into categories of low or high-frequency testers (13, 14). Upon a positive HIV test, individuals were considered diagnosed and could be retained in care. The probability of opting out of care was race-dependent (13, 15). Individuals not retained in care follow the disease progression of untreated individuals.

Table 6. HIV Disease, Screening, and Treatment Parameters

Parameters	Value	Source
**Time individuals spend in the acute phase of HIV infection**	52 days	(16)
**Weibull model parameters for the time until AIDS**		(17)
Beta	1.92934	
Lambda	0.00867	
**Testing frequency categories**		(14)
Never	20.6%	
High frequency	64.3%	
Low frequency	15.1%	
**Testing rate for the high frequency category**	0.00509	(14)
**Testing rate for the low frequency category**	0.00061	
**Proportion of MSM who remained in care upon HIV diagnosis**		
Non-Black MSM	0.40000	(15)
Black MSM	0.31596	(13, 15)
**Monthly probability for unsuppressed individuals to get suppressed (median 3 months)**	0.29289	(15)
**Monthly probability for virologic rebound non-Black population**	0.09763	(15)
**Monthly probability for virologic rebound Black population**	0.15771	(15) (13)

Abbreviation: MSM = men who have sex with men; HIV = human immunodeficiency virus; AIDS = acquired immune deficiency syndrome.

Supplement S4. Details of Pre-exposure prophylaxis (PrEP) Component

Real-world data on PrEP uptake and adherence distributions were obtained from the US PrEP Demonstration Project (PrEP Demo) (18, 19). The PrEP Demo project was a prospective open-label cohort study assessing PrEP delivery in sexually transmitted disease clinics in San Francisco, Miami and Washington, DC. The PrEP Demo project showed a significant association between ethnicity and sexual risk behavior with PrEP uptake and adherence.

PrEP's efficacy was linked to PrEP's adherence according to data reported in Figure 2 of a study by Grant et al. (20). Differential effectiveness as a function of adherence, was derived from a relationship between adherence (pills/week) and tenofovir diphosphate (TVF-DP) levels in the blood (PrEP Demo Project) (21) and a relationship between blood TVF-DP levels and HIV-1 incidence, as measured in the iPrEx-OLE study. The iPrEx-OLE study is a 72-week open-label extension to the iPrEx, ATN 082 and US Safety studies (20).

Table 7. Log-linear regression model for the uptake of PrEP

Covariate	Covariate Coefficients	Covariate Value	Source
Age (per 10-year increase)	0.039	Age/10	(18, 19)
Race-Black	-0.174	0 or 1, depending on the individual	
Race-Hispanic	-0.030	0 or 1, depending on the individual	
Race-Other	-0.167	0 or 1, depending on the individual	
2–5 episodes of anal sex with HIV+ partner during the last 12 months	0.157	0 or 1, depending on the individual	
>5 episodes of anal sex with HIV+ partner during the last 12 months	0.199	0 or 1, depending on the individual	
Prior PrEP awareness	0.445	0.589	
Site-DC	0.285	0.168	
Site-Miami	0.425	0.253	
Education level (> high school)	0.086	0.826	
2–5 episodes of male condom-less anal sex during the last 12 months	0.049	0 or 1, depending on the individual	
>5 episodes of male condom-less anal sex during the last 12 months	0.122	0 or 1, depending on the individual	
HIV risk perception	0.068	0.75	
Referral status- Clinic-referral or Self-referral	0.392	0.376	
Intercept	-1.220		

Abbreviation: HIV = human immunodeficiency virus; PrEP = Pre-exposure prophylaxis.

Table 8. Model for adherence to PrEP

Covariate	Covariate Coefficients	Covariate Value	Source
Race-Black	-1.273	0 or 1, depending on the individual	(19)
Race-Hispanic	-0.211	0 or 1, depending on the individual	
Race-Other	-0.606	0 or 1, depending on the individual	
Living situation (Rent or own housing)	0.703	0.2313	
Site-DC	0.077	0.3265	
Site-Miami	-1.139	0.3231	
Number of condom-less receptive anal sex (if ≥2)	0.599	Calculated for each individual	
Intercept	1.579		

Table 9. PrEP gaps and permanent discontinuations

Parameter	Value	Source
Proportion of PrEP users who discontinue	0.129	(19)
Proportion of PrEP users who would have gaps in taking PrEP	0.028	
PrEP discontinuation (gap) rate among individuals identified to discontinue (have gaps for) PrEP use	0.004	
Mean duration of PrEP gap (days)	65	

Abbreviation: PrEP = Pre-exposure prophylaxis.

Supplement S5. Model Calibration and Validation

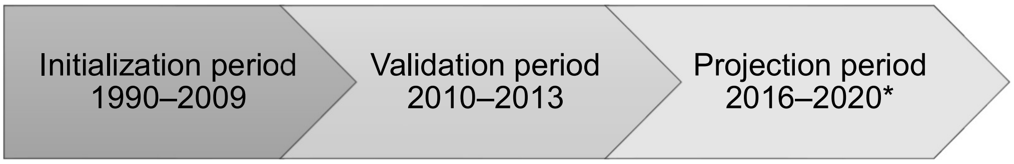

*Though the model validation period ended in 2013, we presented results for 2016–2020 instead of 2014–2020.

AnyLogic software provided us with tools to calibrate our model to historic data available for 2010 through 2013. During the initialization period, the network of sexual partnerships was created, and HIV disease was introduced and transmitted within the sexual partnership network of the MSM. The initial distribution of race and age and the distribution for the number of partners in regular partnerships were adjusted through an iterative calibration process during the initialization period to create the appropriate number of HIV transmissions over time and eventually represent the observed prevalence of HIV during the validation period. The projection period started once the status-quo of the MSM population and HIV epidemic among them were representative of historic data during the validation period.

Supplement S6. Model Validation Results

Figure 1.Simulated and reported HIV prevalence among the MSM population in 2010–2013.

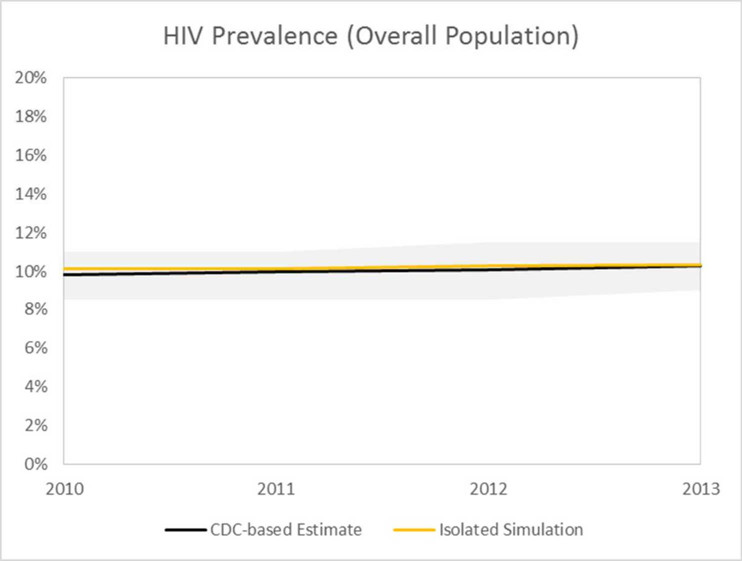

Figure 2. Simulated and reported HIV prevalence among the Black MSM population in 2010–2013.

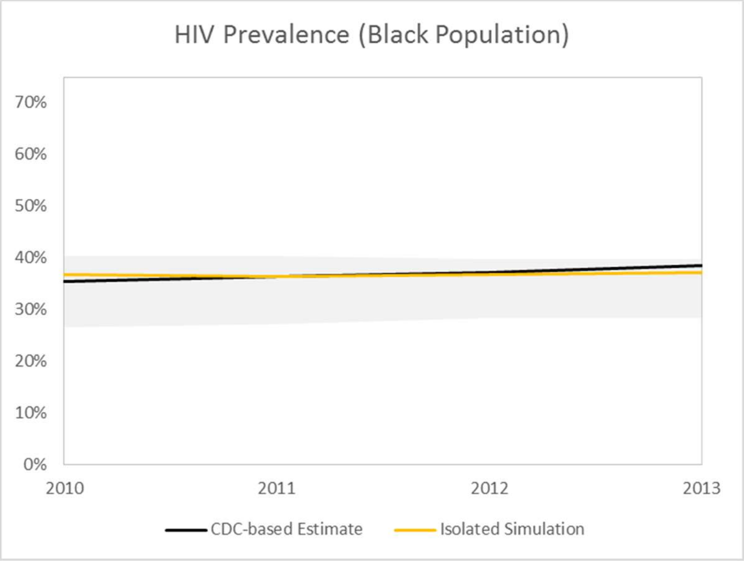

Figure 3. Simulated and reported HIV prevalence among the White MSM population in 2010–2013.

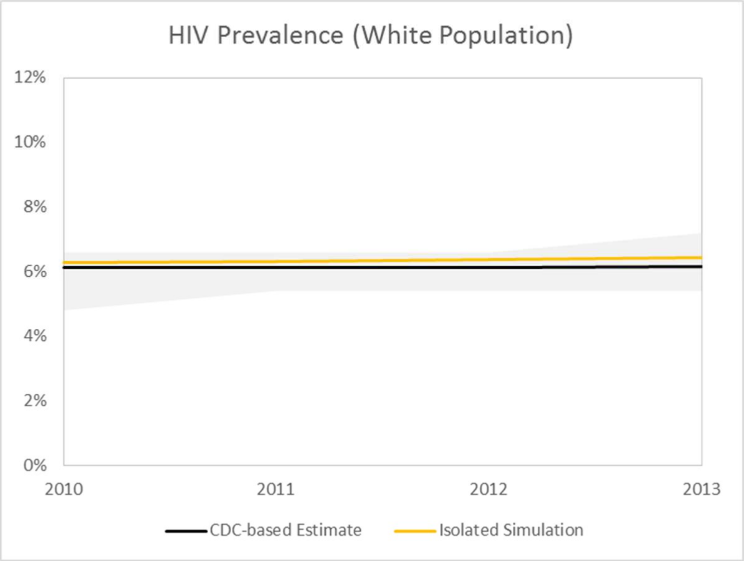

Figure 4. Simulated and reported HIV prevalence among the Hispanic MSM population in 2010–2013.

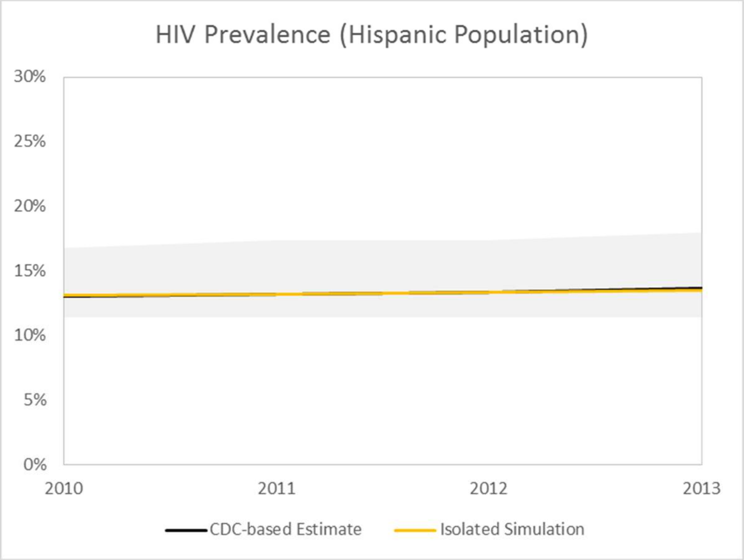

Notes: Figures present the total number of persons living with HIV (diagnosed and undiagnosed). The prevalence of HIV among MSM for each race was calculated according to the following references: CDC HIV Atlas (22), United States Census and a study by Purcell et al. (23) (assuming 3.9% of MSM in United States male population).

References

1. EXPLORE. Data on file. 2016.
2. Lieb S, Fallon SJ, Friedman SR, Thompson DR, Gates GJ, Liberti TM, et al. Statewide estimation of racial/ethnic populations of men who have sex with men in the U.S. Public health reports (Washington, DC : 1974). 2011;126(1):60-72.
3. Newcomb ME, Ryan DT, Garofalo R, Mustanski B. Race-based sexual stereotypes and their effects on sexual risk behavior in racially diverse young men who have sex with men. Arch Sex Behav. 2015;44(7):1959-68.
4. Wei C, Raymond HF. Preference for and maintenance of anal sex roles among men who have sex with men: sociodemographic and behavioral correlates. Arch Sex Behav. 2011;40(4):829-34.
5. Siegler AJ, Sullivan PS, Khosropour CM, Rosenberg ES. The role of intent in serosorting behaviors among men who have sex with men sexual partnerships. J Acquir Immune Defic Syndr. 2013;64(3):307-14.
6. Siegler AJ, Sullivan PS, Khosropour CM, Rosenberg ES. The role of intent in serosorting behaviors among MSM sexual partnerships. Journal of acquired immune deficiency syndromes (1999). 2013;64(3).
7. Patel P, Borkowf CB, Brooks JT, Lasry A, Lansky A, Mermin J. Estimating per-act HIV transmission risk: a systematic review. Aids. 2014;28(10):1509-19.
8. Hollingsworth TD, Anderson RM, Fraser C. HIV-1 transmission, by stage of infection. The Journal of infectious diseases. 2008;198(5):687-93.
9. Hoenigl M, Green N, Mehta SR, Little SJ. Risk Factors for Acute and Early HIV Infection Among Men Who Have Sex With Men (MSM) in San Diego, 2008 to 2014: A Cohort Study. Medicine. 2015;94(30):e1242.
10. Smith DK, Herbst JH, Zhang X, Rose CE. Condom effectiveness for HIV prevention by consistency of use among men who have sex with men in the United States. J Acquir Immune Defic Syndr. 2015;68(3):337-44.
11. Rodger A, Bruun T, Cambiano V, Vernazza P, Estrada V, Lunzen JV, et al., editors. HIV Transmission Risk Through Condomless Sex If HIV+ Partner On Suppressive ART: PARTNER Study. CROI; 2014.
12. Pines HA, Gorbach PM, Weiss RE, Shoptaw S, Landovitz RJ, Javanbakht M, et al. Sexual risk trajectories among MSM in the United States: implications for pre-exposure prophylaxis delivery. J Acquir Immune Defic Syndr. 2014;65(5):579-86.
13. Millett GA, Peterson JL, Flores SA, Hart TA, Jeffries WLt, Wilson PA, et al. Comparisons of disparities and risks of HIV infection in black and other men who have sex with men in Canada, UK, and USA: a meta-analysis. Lancet (London, England). 2012;380(9839):341-8.
14. Centers for Disease Control and Prevention. Morbidity and Mortality Weekly Report (MMWR). 2013;62(47).
15. HIV Surveillance Report. Centers for Disease Control and Prevention; 2008.
16. Suthar AB, Granich RM, Kato M, Nsanzimana S, Montaner JS, Williams BG. Programmatic Implications of Acute and Early HIV Infection. The Journal of infectious diseases. 2015;212(9):1351-60.

17. Time from HIV-1 seroconversion to AIDS and death before widespread use of highly-active antiretroviral therapy: a collaborative re-analysis. Collaborative Group on AIDS Incubation and HIV Survival including the CASCADE EU Concerted Action. Concerted Action on SeroConversion to AIDS and Death in Europe. Lancet (London, England). 2000;355(9210):1131-7.
18. Cohen SE, Vittinghoff E, Bacon O, Doblecki-Lewis S, Postle BS, Feaster DJ, et al. High interest in preexposure prophylaxis among men who have sex with men at risk for HIV infection: baseline data from the US PrEP demonstration project. J Acquir Immune Defic Syndr. 2015;68(4):439-48.
19. Liu AY, Cohen SE, Vittinghoff E, Anderson PL, Doblecki-Lewis S, Bacon O, et al. Preexposure Prophylaxis for HIV Infection Integrated With Municipal- and Community-Based Sexual Health Services. JAMA Intern Med. 2016;176(1):75-84.
20. Grant RM, Anderson PL, McMahan V, Liu A, Amico KR, Mehrotra M, et al. Uptake of pre-exposure prophylaxis, sexual practices, and HIV incidence in men and transgender women who have sex with men: a cohort study. The Lancet Infectious diseases. 2014;14(9):820-9.
21. Cohen SE, Vittinghoff E, Anderson PL, Doblecki-Lewis S, Bacon O, Chege W, et al., editors. Implementation of PrEP in STD Clinics and a Community Health Center: High Uptake and Drug Levels among MSM in the Demo Project. CROI; 2014.
22. NCHHSTP AtlasPlus. Centers for Disease Control and Prevention; 2017 [cited 17 June 2018] Available from: https://www.cdc.gov/nchhstp/atlas/index.htm.
23. Purcell D, Johnson C, Lansky A, Prejean J, Stein R, Denning P, et al., editors. Calculating HIV and syphilis rates for risk groups: estimating the national population size of men who have sex with men. National STD Prevention Conference; 2010.
